# Staged Preoperative Embolization Followed by ICG-Guided Parenchyma-Sparing Resection of Pulmonary Sequestration: A Case Report

**DOI:** 10.3390/jcm15197635

**Published:** 2026-10-02

**Authors:** Giuseppe Mangiameli, Debora Brascia, Giuseppe Ferrillo, Giuseppe Marulli

**Affiliations:** 1 Division of Thoracic Surgery, IRCCS Humanitas Research Hospital, Via Manzoni 56, Rozzano, 20089 Milan, Italy; debora.brascia@hunimed.eu (D.B.);; 2 Department of Biomedical Sciences, Humanitas University, Via Rita Levi Montalcini 4, Pieve Emanuele, 20072 Milan, Italy; 3 Department of Radiology, IRCCS Humanitas Research Hospital, Via Manzoni 56, Rozzano, 20089 Milan, Italy

**Keywords:** pulmonary sequestration, endovascular embolization, indocyanine green, fluorescence-guided surgery, perfusion imaging

## Abstract

Pulmonary sequestration is a rare congenital malformation supplied by an aberrant systemic artery, making parenchyma-sparing resection technically challenging because safe vascular control and accurate identification of the resection plane are both required. Indocyanine green (ICG) fluorescence has been used to delineate the perfusion boundary between sequestrated and normal lung, whereas preoperative embolization has traditionally been performed to reduce the risk of intraoperative bleeding. We describe a staged approach in which selective coil embolization of the aberrant feeding artery, performed one day before surgery, was followed by video-assisted fluorescence-guided wedge resection. Preoperative embolization established devascularization before surgery, while subsequent ICG fluorescence provided clear real-time delineation of the perfusion boundary, enabling tailored parenchyma-sparing resection. Although this observation derives from a single case and does not support routine preoperative embolization, it raises the hypothesis that the interval between embolization and surgery may influence the physiological conditions under which subsequent fluorescence-guided perfusion assessment is performed. This potential effect requires confirmation through quantitative fluorescence assessment and comparative studies.

## 1. Introduction

Pulmonary sequestration is a rare congenital malformation consisting of nonfunctional lung parenchyma supplied by an aberrant systemic artery and lacking normal communication with the tracheobronchial tree. Although surgical resection is commonly performed to prevent recurrent infection, hemoptysis, and other potentially serious complications, management of asymptomatic pulmonary sequestration remains individualized and should take into account clinical presentation, anatomical characteristics, operative risk, and patient preference. However, surgery remains technically demanding because it requires secure control of the aberrant systemic vessel together with precise delineation of the boundary between sequestrated and normally perfused lung, particularly when parenchyma-sparing resection is planned. Failure to accurately define this boundary may result in either incomplete excision of the sequestration or unnecessary sacrifice of healthy functional lung tissue [1,2,3,4,5].

Over the past decade, thoracic surgery has progressively evolved toward lung-preserving strategies whenever technically feasible. Although lobectomy has historically been regarded as the standard treatment for pulmonary sequestration, growing experience with minimally invasive segmentectomy, subsegmentectomy, and non-anatomical wedge resection has demonstrated that parenchyma-sparing procedures represent effective alternatives in carefully selected patients with localized disease. These approaches preserve postoperative pulmonary function while ensuring complete excision and have become increasingly feasible owing to advances in preoperative imaging, three-dimensional reconstruction, and intraoperative navigation. Recent reports have demonstrated that complex thoracoscopic and uniportal sublobar resections can be safely performed for intralobar pulmonary sequestration when meticulous preoperative planning is combined with accurate intraoperative definition of the resection plane [6,7].

This evolution has paralleled the widespread adoption of fluorescence-guided thoracic surgery. Indocyanine green (ICG) fluorescence imaging is now routinely used during anatomical segmentectomy, providing real-time visualization of pulmonary perfusion and facilitating identification of intersegmental planes. More recently, this concept has been extended to pulmonary sequestration, where intravenous ICG administered after interruption of the aberrant systemic artery enables clear visualization of the boundary between healthy and sequestrated lung. A retrospective clinical series reported clear fluorescence-based delineation of the boundary between normally perfused and sequestrated lung in all six patients undergoing intraoperative ICG imaging, supporting the feasibility of this approach for parenchyma-sparing resection [3,4,5,6]. Likewise, near-infrared fluorescence imaging has been successfully applied during robotic complex segmentectomy, further supporting the integration of functional perfusion mapping into increasingly conservative thoracic surgical procedures [8].

Collectively, these developments reflect the broader transition toward image-guided precision thoracic surgery, in which real-time functional information complements conventional anatomical dissection and supports increasingly individualized surgical planning. Nevertheless, successful fluorescence-guided resection of pulmonary sequestration still depends on effective control of the aberrant systemic arterial supply.

To facilitate surgery for pulmonary sequestration, two adjunctive strategies have progressively gained acceptance. Preoperative endovascular embolization has been adopted in selected patients to reduce the risk of intraoperative hemorrhage through occlusion of the systemic feeding artery [1,2], whereas ICG fluorescence imaging provides real-time identification of the perfusion boundary, thereby facilitating tailored parenchyma-sparing resection [3,4,5,6]. Although these techniques are increasingly employed in modern thoracic surgery, they have generally been regarded as complementary yet independent approaches, each addressing a distinct technical challenge.

Beyond its established role in vascular control, preoperative embolization may also influence regional pulmonary perfusion before surgery and thereby modify the physiological environment in which intraoperative fluorescence imaging is performed. We hypothesized that staged interruption of the systemic arterial inflow approximately 24 h before surgery might influence the physiological conditions under which subsequent intraoperative perfusion assessment is performed. To our knowledge, no previous study has specifically investigated whether staged preoperative embolization may modify the physiological conditions under which fluorescence-guided surgery is performed. We therefore report our experience with selective coil embolization performed the day before surgery followed by ICG-guided thoracoscopic wedge resection, and discuss the hypothesis that the interval between embolization and surgery may influence subsequent fluorescence-based perfusion assessment.

Ethical statement: Written informed consent for publication was obtained from the patient. Ethical approval was not required according to institutional policy.

## 2. Case Presentation

A 61-year-old asymptomatic man with incidentally diagnosed intralobar pulmonary sequestration of the right lower lobe was referred to our institution for surgical management. Contrast-enhanced computed tomography demonstrated a well-defined sequestration supplied by a single aberrant systemic feeding artery measuring 5.65 mm in diameter, originating from the descending thoracic aorta and coursing within the inferior pulmonary ligament toward the right lower lobe. Venous drainage occurred through the right inferior pulmonary vein, with no additional arterial or venous vascular anomalies identified. Selective angiography subsequently confirmed the single systemic feeding artery and demonstrated multiple distal branches supplying the sequestrated parenchyma.

Because the lesion was confined to a limited portion of the lower lobe, complete excision appeared feasible without anatomical pulmonary resection. Given the patient’s absence of symptoms, the localized extent of the lesion, and the objective of maximizing preservation of functional lung parenchyma, a parenchyma-sparing resection was considered appropriate. Preoperative embolization was selected to obtain secure control of the 5.65-mm systemic feeding artery before thoracoscopic resection and thereby minimize the risk of intraoperative hemorrhage. The presence of a single, clearly identifiable feeding artery with multiple distal branches made selective endovascular occlusion technically feasible. Following multidisciplinary discussion between thoracic surgeons and interventional radiologists, a staged hybrid approach consisting of preoperative selective endovascular embolization followed by fluorescence-guided thoracoscopic atypical (wedge) resection was therefore selected rather than anatomical segmentectomy or lobectomy.

Preoperative pulmonary function was well preserved (FEV_1_ 124% predicted; DLCO 81% predicted), further supporting a parenchyma-sparing surgical strategy.

The clinical course from diagnosis through treatment and follow-up is summarized in Table 1.

## 3. Surgical Technique

### 3.1. Preoperative Planning and Endovascular Embolization

**Figure 1:**
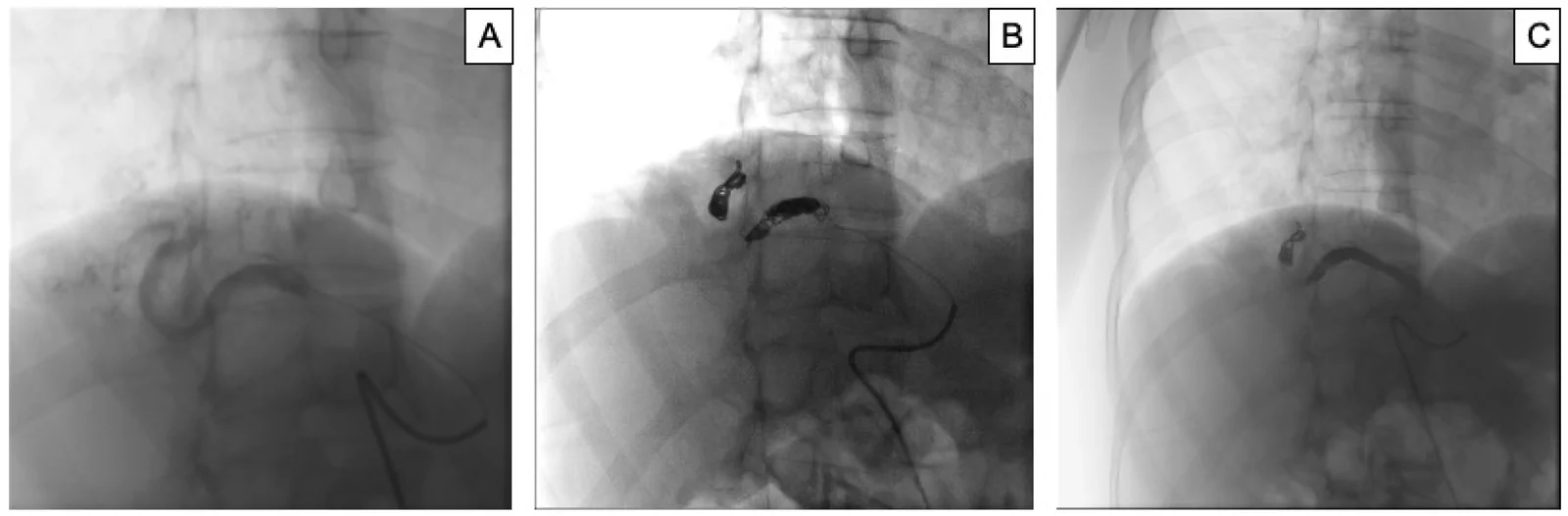
Preoperative embolization of the aberrant systemic feeding artery. (**A**) Angiography showing the feeding artery from the descending thoracic aorta. (**B**) Coil deployment. (**C**) Post-embolization angiography confirming occlusion.

Preoperative contrast-enhanced computed tomography was used to define the origin, course, and caliber of the aberrant systemic feeding artery and to plan both the endovascular and surgical procedures. The day before surgery, selective endovascular embolization was performed through a common femoral arterial approach. After superselective catheterization of the feeding vessel, complete occlusion was achieved using detachable microcoils (Embold^®^ Soft, Boston Scientific Corporation, Marlborough, MA, USA, 5- and 6-mm), followed by additional packing coils (Embold^®^ Packing, Boston Scientific Corporation, Marlborough, MA, USA), resulting in complete angiographic exclusion of the systemic arterial supply (Figure 1). The coils were intentionally deployed distal to the anticipated surgical division site, leaving a proximal coil-free segment of the aberrant artery to allow subsequent application of the vascular stapler. No embolization-related adverse events occurred, including significant pain, pulmonary infarction, pleural reaction, coil migration, or access-site complications.

### 3.2. Thoracoscopic Resection

Approximately 24 h after embolization, pulmonary resection was performed through a video-assisted mini-thoracotomy. This interval was selected pragmatically within the planned staged workflow and was not based on a predefined physiological or fluorescence-related endpoint. The embolized feeding artery was carefully dissected, isolated, and divided using an endoscopic vascular stapler (EndoGIA™, Medtronic, Minneapolis, MN, USA, vascular reload). In our experience, prior coil embolization did not interfere with vascular stapling or safe transection of the vessel, and no significant intraoperative bleeding occurred.

### 3.3. Fluorescence-Guided Parenchymal Resection

**Figure 2:**
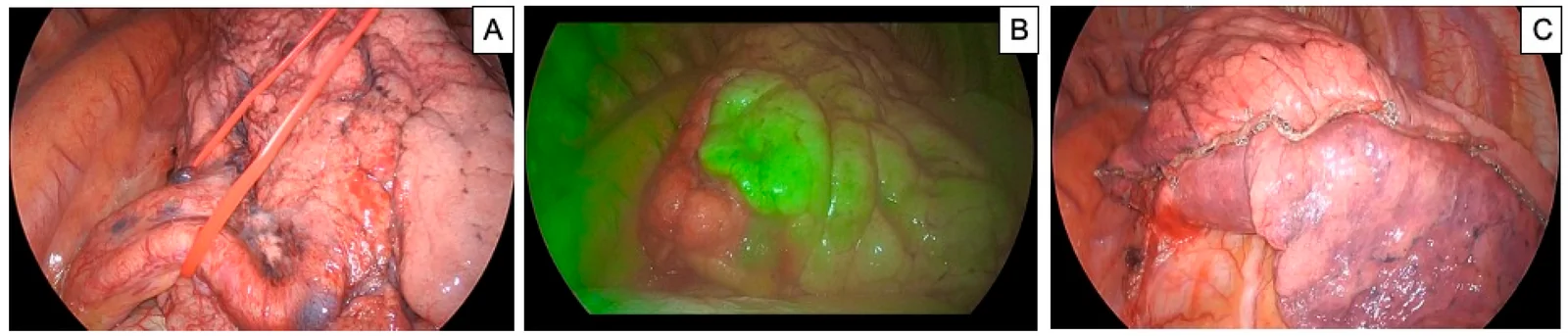
Intraoperative near-infrared fluorescence imaging after staged preoperative embolization (**A**) White-light view with ill-defined boundaries. (**B**) Near-infrared imaging showing clear demarcation between fluorescent normal lung and non-fluorescent sequestration. (**C**) Fluorescence-guided tailored resection.

Following complete vascular control, indocyanine green (0.1 mg/kg) was administered intravenously. Near-infrared fluorescence imaging was performed using a 4K thoracoscopic imaging system with integrated near-infrared capability (Stryker, Kalamazoo, MI, USA). A clear fluorescence demarcation between the normally perfused lung and the devascularized sequestration became visible approximately 15–20 s after ICG administration. The fluorescence-defined boundary was consistent with the extent of the sequestration identified on preoperative CT. The fluorescence-defined demarcation line was subsequently used to perform a tailored atypical (wedge) resection, allowing complete excision of the sequestrated parenchyma while maximizing preservation of the surrounding functional lung (Figure 2). Total operative time was 83 min, with estimated blood loss of <50 mL. The postoperative course was uneventful. Postoperative chest radiography demonstrated complete lung re-expansion, and the chest tube was removed on postoperative day 1. The patient was discharged on postoperative day 2. Histopathological examination confirmed the diagnosis of pulmonary sequestration, with complete excision and uninvolved resection margins. At 5-month clinical follow-up, the patient remained asymptomatic and had resumed regular physical and sporting activities.

## 4. Discussion

Parenchyma-sparing resection of pulmonary sequestration requires complete excision of the sequestrated lung while preserving as much functional pulmonary parenchyma as possible. Although secure control of the aberrant systemic artery remains the cornerstone of safe surgical treatment, precise identification of the boundary between sequestrated and normally perfused lung is equally important to avoid either incomplete resection or unnecessary sacrifice of healthy tissue. This principle has become increasingly relevant with the widespread adoption of minimally invasive thoracic surgery and the progressive shift toward anatomical and non-anatomical lung-preserving procedures for benign localized diseases. In appropriately selected patients, segmentectomy, subsegmentectomy, or wedge resection can preserve postoperative pulmonary function without compromising surgical radicality, provided that the entire sequestrated parenchyma is accurately identified and completely removed [6,7].

Preoperative endovascular embolization and intraoperative indocyanine green (ICG) fluorescence imaging have each been proposed to facilitate this objective, although they have traditionally been regarded as independent adjunctive techniques addressing different aspects of the procedure. To date, hybrid approaches have primarily focused on the role of embolization in reducing the risk of catastrophic intraoperative hemorrhage, particularly in patients with large-caliber aberrant arteries, complex vascular anatomy, or minimally invasive surgical approaches. Previous reports have also emphasized the importance of multidisciplinary planning to optimize the embolization strategy and avoid interference between embolization coils and subsequent vascular stapling or ligation [1,2]. In our case, close collaboration with the interventional radiology team enabled complete occlusion of the feeding artery while preserving straightforward surgical division of the vessel, and no technical difficulties related to the previously deployed coils were encountered.

In parallel, increasing evidence supports the use of ICG fluorescence imaging for intraoperative identification of the perfusion boundary in pulmonary sequestration. As summarized in Table 2, the published literature has progressively evolved from isolated case reports demonstrating the technical feasibility of fluorescence-guided surgery to larger clinical experiences supporting its reproducibility during minimally invasive parenchyma-sparing resection. Early reports established the feasibility of this approach in individual patients, whereas subsequent studies confirmed its reproducibility in routine clinical practice. In a retrospective series of 17 patients, Nakanishi and colleagues used intraoperative ICG imaging in six patients, in whom fluorescence imaging consistently delineated the interface between sequestrated and normally perfused lung and supported tailored parenchyma-sparing resection. They also highlighted that fluorescence guidance may reduce the uncertainty associated with conventional visual assessment, particularly when the anatomical limits of the sequestration are poorly defined. Similarly, Motohashi and colleagues provided important physiological insights by demonstrating that the sequestrated lung exhibits delayed and heterogeneous fluorescence before interruption of the aberrant systemic artery, whereas fluorescence disappears completely after interruption of the systemic inflow, confirming that the observed fluorescence pattern reflects regional pulmonary perfusion rather than anatomical boundaries alone [5,6].

The novelty of our approach does not lie in the individual use of preoperative embolization or ICG fluorescence, nor in their combined use, which has already been reported. In most previous experiences, interruption of the aberrant systemic artery was performed intraoperatively shortly before fluorescence assessment. Notably, Nakanishi and colleagues also reported two patients who underwent coil embolization the day before surgery followed by intraoperative ICG imaging; however, the potential physiological implications of the interval between embolization and fluorescence assessment were not specifically investigated [6]. In the present report, we specifically focus on the hypothesis that a preceding interval of devascularization may influence the physiological conditions under which subsequent fluorescence-based perfusion assessment is performed. Thus, rather than proposing the staged combination itself as novel, this case provides a hypothesis-generating physiological rationale for investigating the potential interaction between preoperative vascular modulation and subsequent ICG-guided parenchyma-sparing resection.

Although our observation derives from a single patient and should be considered hypothesis-generating, this temporal interval may have relevant physiological implications. Following embolization, the sequestrated lung is progressively excluded from the systemic circulation, allowing the acute hemodynamic changes induced by vessel occlusion to stabilize before surgery. We hypothesize that, when ICG is administered intraoperatively after definitive vascular division, the preceding interval of devascularization might influence the perfusion conditions under which fluorescence imaging is performed. Whether this translates into clearer discrimination between perfused and non-perfused lung tissue cannot be determined from the present case and would require quantitative and comparative evaluation.

Importantly, this proposed mechanism should not be interpreted as evidence that preoperative embolization directly enhances ICG fluorescence or increases fluorescence intensity. The optical properties of ICG remain unchanged, and our study does not provide quantitative fluorescence measurements. Rather, we hypothesize that the interval between embolization and surgery may influence the hemodynamic conditions under which fluorescence imaging is subsequently performed. Whether this has any measurable effect on intraoperative perfusion mapping remains unknown. From a practical perspective, the proposed staged strategy does not increase the technical complexity of fluorescence-guided surgery. Endovascular embolization is already performed in selected patients to reduce the risk of hemorrhage, particularly when the aberrant artery is large, fragile, or technically difficult to control thoracoscopically. In these patients, performing embolization approximately one day before surgery requires no modification of the standard intraoperative fluorescence protocol. After surgical division of the embolized vessel, intravenous ICG is administered according to the conventional technique, allowing surgeons to benefit from both secure vascular control and real-time perfusion guidance during parenchymal resection.

From a broader surgical perspective, our experience highlights the evolving role of multimodal image-guided strategies in thoracic surgery. Rather than viewing embolization and fluorescence imaging as separate technical adjuncts, these modalities may be considered complementary components of a single workflow aimed at maximizing procedural safety while preserving functional lung tissue. Beyond pulmonary sequestration, the concept of combining preoperative vascular modulation with intraoperative functional imaging may have broader implications for precision thoracic surgery. As minimally invasive and robotic techniques continue to evolve, real-time assessment of tissue perfusion is increasingly incorporated into intraoperative decision-making. The integration of advanced imaging, three-dimensional planning, endovascular interventions, and fluorescence guidance may ultimately enable increasingly individualized lung-preserving procedures tailored not only to anatomical characteristics but also to regional pulmonary physiology.

Although the present report focuses on pulmonary sequestration, the underlying concept may have broader implications. Similar strategies could potentially be explored whenever regional pulmonary perfusion needs to be accurately identified before limited pulmonary resection. The combination of staged vascular modulation and real-time fluorescence imaging may therefore represent an additional step toward physiology-guided thoracic surgery, in which surgical decision-making is increasingly supported by functional rather than purely anatomical information. While this concept remains speculative, it is consistent with the ongoing evolution of minimally invasive thoracic surgery toward increasingly image-guided and physiology-based procedures.

Several limitations should be acknowledged. First, this report describes a single clinical case and therefore cannot establish a causal relationship between staged embolization and improved fluorescence-guided identification of the resection plane. Second, no quantitative fluorescence analysis was performed. Consequently, it remains unknown whether preoperative embolization influences objective fluorescence parameters, such as fluorescence intensity, contrast, or time to maximal enhancement. Third, because no control patient underwent immediate intraoperative division of the feeding artery, direct comparison between the two strategies was not possible. Finally, the proposed physiological explanation should be regarded as a hypothesis derived from intraoperative observation rather than a demonstrated mechanism. Moreover, the present findings should not be interpreted as supporting routine preoperative embolization in all patients with pulmonary sequestration, but rather as providing a rationale for further investigation in carefully selected cases. Prospective studies incorporating quantitative near-infrared fluorescence assessment and standardized perfusion analysis will be required to determine whether staged embolization provides measurable advantages over conventional intraoperative vascular control. Overall, our experience demonstrates the technical feasibility of combining staged preoperative embolization with subsequent fluorescence-guided parenchyma-sparing resection. Whether the preceding period of devascularization influences subsequent fluorescence-based assessment of pulmonary perfusion remains unknown. Nevertheless, this observation provides a hypothesis-generating framework for investigating the potential interaction between preoperative vascular modulation and intraoperative fluorescence imaging.

## 5. Conclusions

Preoperative embolization has an established role in facilitating vascular control during resection of pulmonary sequestration. This case demonstrates the technical feasibility of combining staged preoperative embolization with subsequent ICG-guided parenchyma-sparing resection. Whether the interval of preoperative devascularization influences subsequent fluorescence-based perfusion assessment remains unknown. The physiological rationale proposed herein should therefore be considered hypothesis-generating and warrants evaluation using quantitative fluorescence analysis and comparative studies.

## Figures and Tables

**Table 1:** Clinical timeline according to CARE reporting principles.

Time Point	Clinical Event
Initial presentation	Incidental diagnosis of asymptomatic intralobar pulmonary sequestration of the right lower lobe
Preoperative assessment	Contrast-enhanced CT demonstrated a localized sequestration supplied by a single 5.65-mm aberrant systemic artery arising from the descending thoracic aorta; venous drainage through the right inferior pulmonary vein
Day −1	Selective endovascular coil embolization of the aberrant systemic feeding artery with complete angiographic occlusion; no procedure-related adverse events
Day 0	Video-assisted parenchyma-sparing wedge resection with intraoperative ICG fluorescence guidance; operative time 83 min; estimated blood loss <50 mL
Postoperative day 1	Chest radiography demonstrated complete lung re-expansion; chest tube removed
Postoperative day 2	Uneventful postoperative recovery; patient discharged
Final pathology	Histopathological confirmation of pulmonary sequestration; complete excision with uninvolved resection margins
5-month follow-up	Patient asymptomatic and clinically well; resumed regular physical and sporting activities

**Table 2:** Published experience with preoperative embolization and indocyanine green (ICG) fluorescence-guided surgery for pulmonary sequestration.

Author	Year	Patients	Embolization	Timing of Embolization	Aberrant Artery (Origin/Size)	ICG	Type of Resection	Main Message
Yamanashi [3]	2018	1	No	-	2 arteries from celiac artery; diameter NR	Yes	Wedge	ICG delineation of sequestration boundary
Motono [4]	2019	1	No	-	Single artery from descending aorta; diameter NR	Yes	Wedge	ICG-guided parenchyma-sparing resection
Motohashi [5]	2021	1	No	-	2 arteries from descending aorta; diameter NR	Yes	Segmentectomy	Hemodynamic assessment with ICG
Nakanishi [1]	2021	1	Yes	Day before surgery	Single artery from descending thoracic aorta; ~4 mm	Yes	Segmentectomy	Hybrid surgery; technical issues related to intravascular coil
Zhou [2]	2018	1	Yes	Same hybrid session	Single artery from abdominal aorta; 4 mm	No	VATS wedge	Embolization to reduce bleeding risk
Nakanishi [6]	2023	17 (6 with ICG)	4/17 overall; 2/6 ICG cases	Day before surgery in ICG cases	Thoracic/abdominal aorta; 2–9 mm in ICG subgroup	6/17	All 6 ICG cases underwent segmentectomy	ICG-guided delineation
Present study	2026	1	Yes	~24 h before surgery	Single artery from descending thoracic aorta; 5.65 mm	Yes	VATS Wedge	Staged embolization hypothesis

NR, not reported; ICG, indocyanine green; VATS, video-assisted thoracic surgery. Available reports are heterogeneous and predominantly consist of case reports or small observational experiences; therefore, this table is intended as a descriptive overview and should not be interpreted as providing comparative evidence.

## Data Availability

The data supporting the findings of this study are available from the corresponding author upon reasonable request.
